# Comparison of gene coverage of mouse oligonucleotide microarray platforms

**DOI:** 10.1186/1471-2164-7-58

**Published:** 2006-03-21

**Authors:** Ricardo A Verdugo, Juan F Medrano

**Affiliations:** 1 Department of Animal Science, University of California Davis, One Shields Avenue, Davis, CA 95616-8521, USA

## Abstract

**Background:**

The increasing use of DNA microarrays for genetical genomics studies generates a need for platforms with complete coverage of the genome. We have compared the effective gene coverage in the mouse genome of different commercial and noncommercial oligonucleotide microarray platforms by performing an in-house gene annotation of probes. We only used information about probes that is available from vendors and followed a process that any researcher may take to find the gene targeted by a given probe. In order to make consistent comparisons between platforms, probes in each microarray were annotated with an Entrez Gene id and the chromosomal position for each gene was obtained from the UCSC Genome Browser Database. Gene coverage was estimated as the percentage of Entrez Genes with a unique position in the UCSC Genome database that is tested by a given microarray platform.

**Results:**

A MySQL relational database was created to store the mapping information for 25,416 mouse genes and for the probes in five microarray platforms (gene coverage level in parenthesis): Affymetrix430 2.0 (75.6%), ABI Genome Survey (81.24%), Agilent (79.33%), Codelink (78.09%), Sentrix (90.47%); and four array-ready oligosets: Sigma (47.95%), Operon v.3 (69.89%), Operon v.4 (84.03%), and MEEBO (84.03%). The differences in coverage between platforms were highly conserved across chromosomes. Differences in the number of redundant and unspecific probes were also found among arrays. The database can be queried to compare specific genomic regions using a web interface. The software used to create, update and query the database is freely available as a toolbox named ArrayGene.

**Conclusion:**

The software developed here allows researchers to create updated custom databases by using public or proprietary information on genes for any organisms. ArrayGene allows easy comparisons of gene coverage between microarray platforms for any region of the genome. The comparison presented here reveals that the commercial microarray Sentrix, which is based on the MEEBO public oligoset, showed the best mouse genome coverage currently available. We also suggest the creation of guidelines to standardize the minimum set of information that vendors should provide to allow researchers to accurately evaluate the advantages and disadvantages of using a given platform.

## Background

The wide use of DNA microarrays to query expression of genes has created the need for updated, consistent and meaningful annotations on the probes included in the microarrays. We refer to gene annotation as a recognizable label or gene id identifying the gene that is targeted by a given probe. Gene ids should be stable, widely used and allow reliable associations among genomic databases. Several microarray annotation systems are available for investigators, aiming to address specific user demands. For instance, the KARMA [1] web server provides periodically updated gene annotations of Keck arrays [2] and Affymetrix^® ^GeneChips^® ^[3], and can also annotate user-provided lists of accession numbers for pair-wise comparisons, even for different species. However, providing a gene list is not always a straight forward process given the large heterogeneity in the format that vendors provide sequence identifiers for probes. For instance, one platform can include identifiers in a Genbank header format such as GB|AY073000.1|AAL60663.1 and others may include different types of ids separated by commas or some other character within a single column. In addition, different sequence identifiers in several columns may be provided by vendors and choosing only one of them may not be the best solution. The Resourcerer database [4] tackles this problem by pre-computing gene annotations on a more exhaustive list of microarrays and oligosets for a number of species [5]. This database is centered on 'tentative consensus' (TC) sequences which are used as gene definitions. TCs group EST sequences that can be aligned and clustered in distinct groups, and these are periodically updated as new ESTs from GenBank become available. Functional annotations on these TCs generate the *Gene Indices *resource available from the TGI website [6]. TCs allow for cross species comparisons through the Tentative Orthologue Groups (TOGs) database. However, Gene Indices are not stable and cross referencing to other genomic databases is not easy. A different approach has been taken by Mattes[7] who created a set of Perl scripts that use UniGene and LocusLink as gene identifiers, providing a more universal gene definition that can be cross referenced with other databases. Unfortunately, the recent shift of NCBI from LocusLink to the Entrez Gene database format [8] has limited the functionality of these scripts and rendered them obsolete. The DRAGON [9] database [10,11], and the DAVID [12] software [13] provide web based services of gene annotation with similar objectives. None of them, however, allows for chromosome or genomic-region specific comparisons of gene coverage.

**Figure 1:**
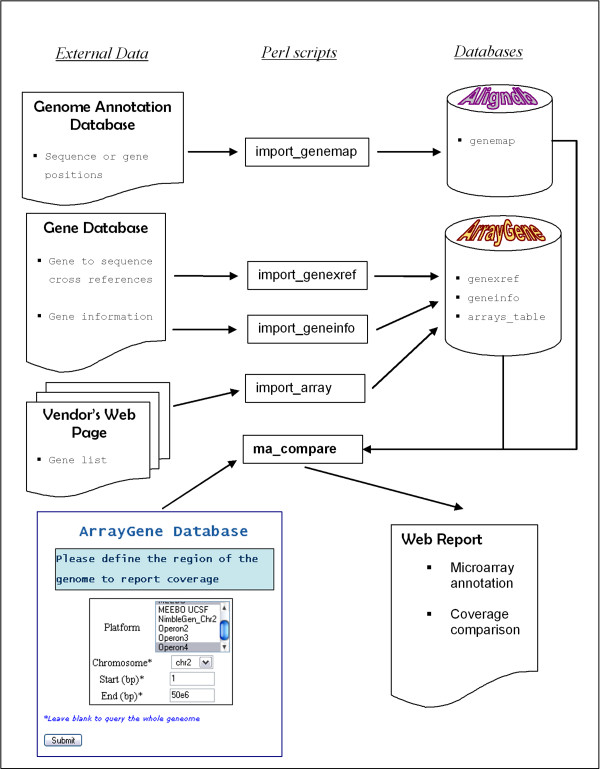
**Flowchart diagram for the construction and query of the *ArrayGene *and *Aligndb *databases**. Perl scripts are used to parse input files from other databases and upload the processed information to the ArrayGene and Aligndb databases. The ma_compare CGI (Common Gateway Interface) script written in Perl is used to process queries through the web and produce online reports for gene coverage.

The objective of this study was to compare gene coverage from currently available whole mouse genome microarrays for any region of the genome. We only used the information about probes provided to researchers by vendors before the purchase of a microarray for the purpose of choosing the platform that best fits their needs. We have developed a platform for microarray annotation that not only provides gene annotations for probes but also genomic positions for tested genes in the mouse genome. Coverage comparisons can be obtained for any genomic region of the last available mouse assembly build. The level of coverage of five whole mouse genome microarrays and four oligosets was compared in the present study. Microarrays and oligosets will be referenced here by the short name provided in Table 3. The results were stored in a relational database that can readily be queried for coverage comparisons based on genome position. Figure 1 shows a flowchart diagram for the databases and methods used for the annotation system and for querying gene coverage comparisons.

## Results

### Number of genes in the genome

**Figure 2:**
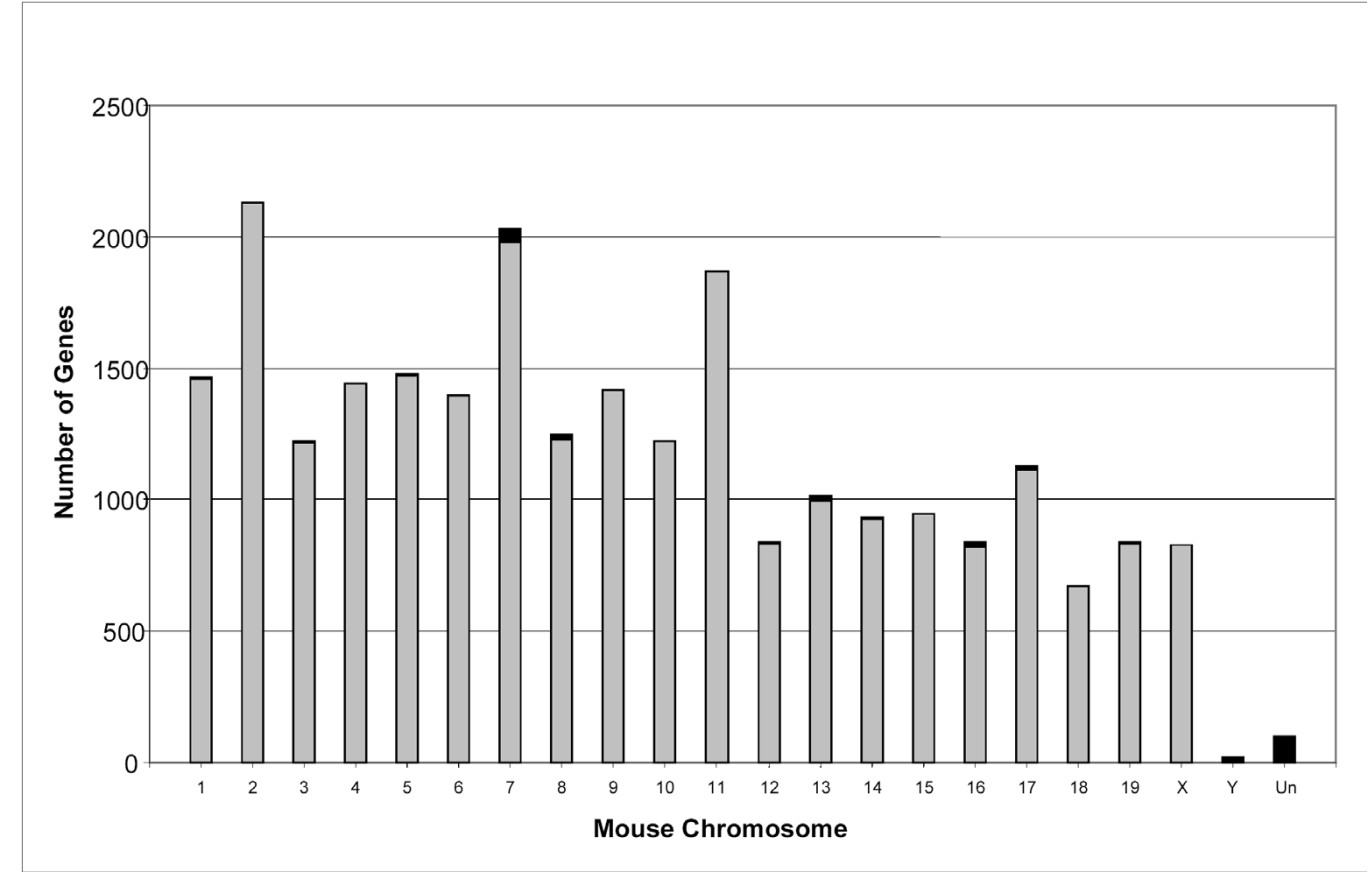
**Distribution of Entrez Genes per chromosome from the UCSC Genome Browser Database**. The fraction of genes mapping to unordered scaffolds is shown in black. Mapped genes are in gray. The last bar represents genes mapping to scaffolds that could not be mapped to any chromosome in the genome.

The total number of genes in the genome was defined as the number of Entrez Genes with a unique genomic position at the UCSC Genome Browser Database [14] (see methods). A total of 198,155 mapped sequences from the Known Gene, RefGene, and mRNA tracks were associated with Entrez Genes. A total of 521 genes could not be used because they are located in unordered scaffolds in Build 35.1 of the mouse genome assembly. Multiple sequence alignments in the genomes were found for a total of 1,766 sequences. For example, the M10062 cDNA aligns with chromosomes 1, 2, 3, 4, 6, 10, 11, 13, 15, 17, 19, X, and Un_random (not chromosome assigned contigs) chromosomes. This cDNA is identified as the *Iap *gene in the Entrez Gene database. This is a retrotransposon that can be found in several chromosomes and does not have a unique position. Genes like this, and other not so extreme cases, cannot be considered in region-specific coverage comparisons and were therefore discarded. Table 2 shows the number of genes that could be assigned to specific position in the genome in each of the source files. The file Mm.gb_cid_lid in Table 1 was used to incorporate associations between sequences and Unigene ids in the *genexref *table. Although we do not use Unigene annotations of probes to identify the targeted genes (see discussion section for explanation) this allowed us to associate Entrez Gene ids with EST accession numbers, which are commonly used in microarray genes lists. A total of 25,416 genes could be found having a unique position in the August 2005 mouse genome assembly (Build 35.1). The distribution of these genes in the genome is shown in Figure 2.

### Microarrays probe annotations

**Figure 3:**
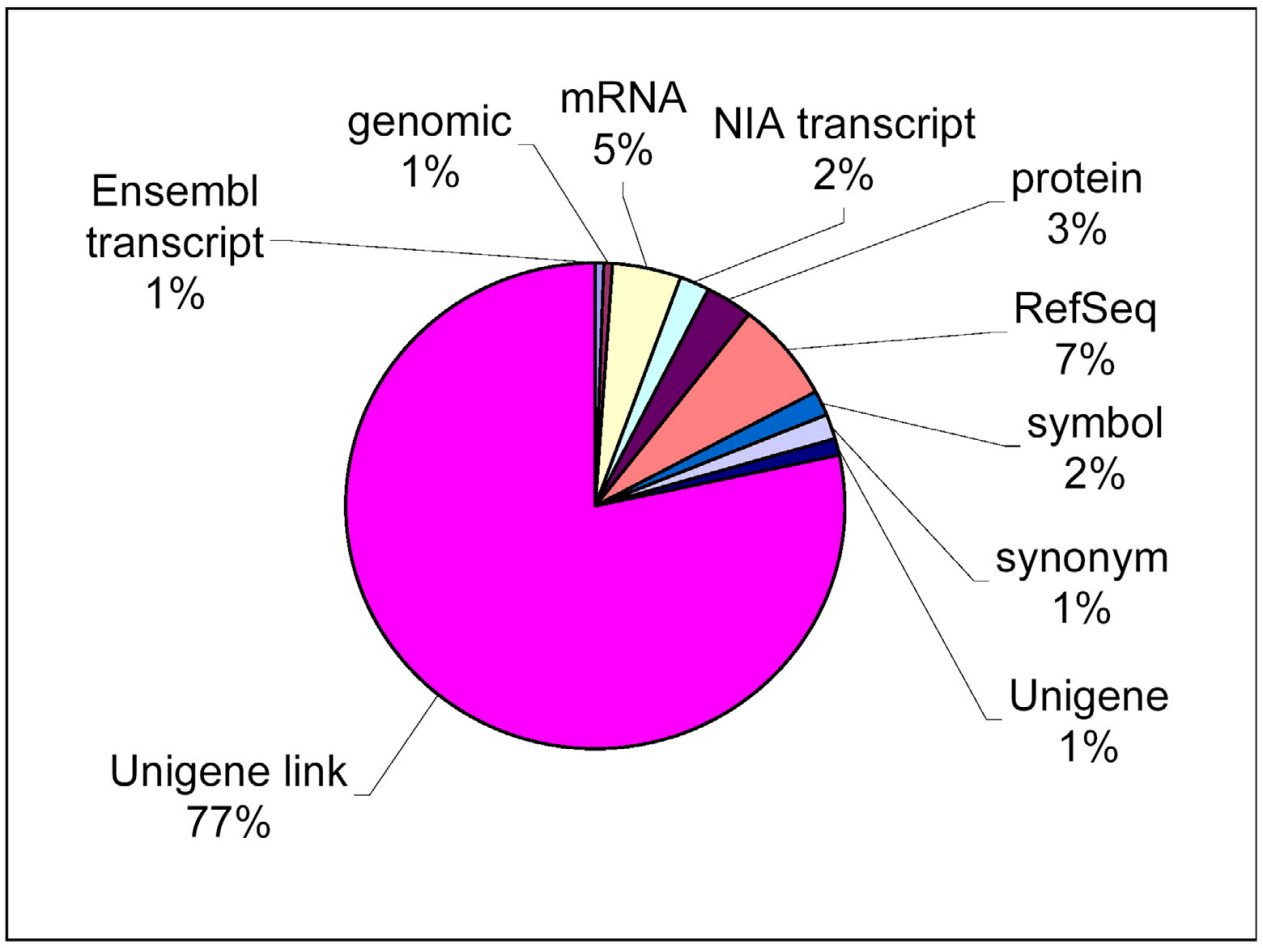
**Sequence Identifiers Associated with Entrez Gene ids in the ArrayGene database**. The mRNA, genomic, and protein classes represent GenBank accession numbers that have been associated with an Entrez Gene by NCBI. RefSeq groups reference mRNAs, genomic and protein sequences. Ensembl transcript are ids from Ensembl. Unigene, symbol, and synonym are gene identifications than have been cross-referenced with an Entrez Gene id by NCBI. Unigene link are sequence accession numbers that have been clustered into Unigene ids that are associated with Entrez Genes. NIA transcripts represent ids of transcripts from the National Institute of Aging mouse Gene Index (V. 4.0) [33].

**Figure 4:**
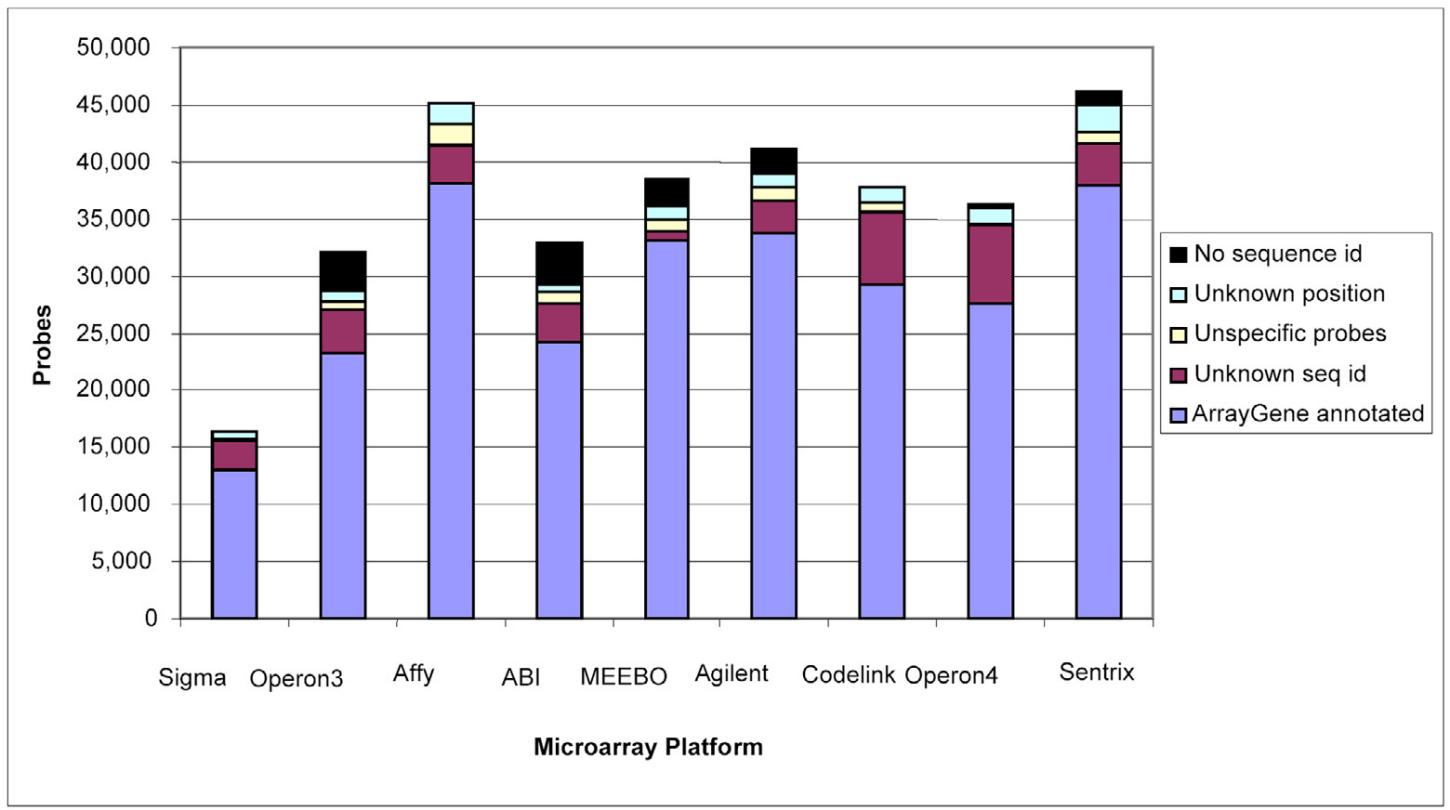
**Efficiency of gene annotation of probes by ArrayGene**. The height of the bars shows total number of probes in the array. No sequence id represents probes for which there was no sequence annotation available in the gene. Unknown position probes are those that the gene is known but it maps to multiple positions in the genome or to unordered scaffolds. Unspecific probes refer to those that can be associated with more than one Entrez Gene. Unknown sequence id refers to probes annotated with identifiers that could not be associated with an Entrez Gene. *ArrayGene *annotated probes are those that could be associated with a single Entrez Gene id that map to a known position in the genome. For the ABI platform, the No Sequence id fraction corresponds to about 4,000 probe targeting genes that have not been annotated by the public effort and are only available from the Celera gene discovery system.

**Figure 5:**
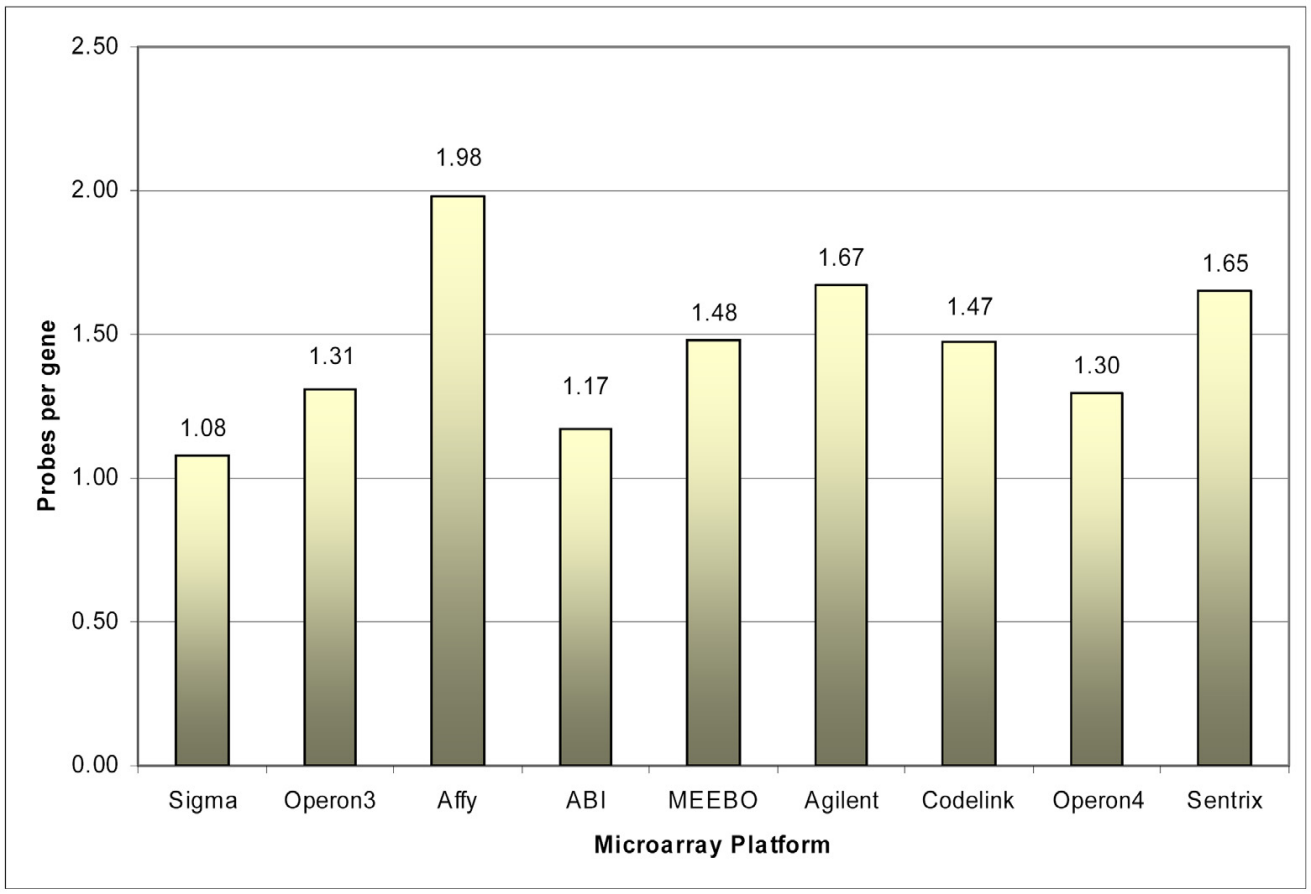
Average number of probes or probe sets that test the same gene in different microarray platforms.

The Resourcerer database provides gene annotations for most of the available microarrays and oligosets for mouse and other organisms [4]. However, database updates are done at four-month intervals, and in our experience, gene annotations change periodically and many Entrez Gene ids in the Resourcerer annotations are obsolete. Microarray vendors also provide gene annotations on their probes, but they vary greatly in the number and quality of the annotations. For instance, ABI is the platform with the largest number of probes annotated with a gene id by the vendor (Table 4). However, these are Celera Genomics^® ^gene identifications and cannot be directly matched to public domain genes. Therefore, we opted for performing our own gene annotation of probes using the most updated information at hand (see methods). Our *genexref *table, in the *ArrayGene *database, stored cross reference information between 765,289 sequence identifiers and 63,175 Entrez Genes. The different kinds of sequence identifiers included in the database are shown in Figure 3. Performing in-house annotations allowed us to discard any probe that could be associated with more than one gene. Probe annotation files, referred to as Genelists, were obtained directly from vendors' websites and they identify the sequences from which oligonucleotide probes where designed. The format and amount of information provided in these lists varied greatly, Affymetrix being the most comprehensive in the number of different annotations. The efficiency of the gene annotation process varied between platforms depending on the amount of sequence identifiers provided by vendors and the level of specificity of the information provided (Figure 4). Specificity is defined here as the number of associations that can be inferred between a probe and Entrez Genes from all the probe annotations provided by the vendor. The gene annotations from the *ArrayGene *system do not include any probe that could be associated with more than one gene nor genes with an uncertain position in the genome. This approach created a more conservative set of gene annotations than those included in the Resourcerer database. In most of the platforms we could not match the number of probes annotated with gene ids by the vendor (Entrez Gene ids, gene symbols, UniGene ids, etc) given our stringent criteria to select a unique gene identifier ("Unknown seq id" in Figure 4). The level of redundancy, i.e. the number of probes hybridizing to the same gene, also varied between platforms (Figure 5), the least redundant being the Sigma platform (1.08 probes per gene on average), though it has the least number of probes. However, the newer ABI platform has a comparable level of redundancy (1.17 probes per gene). The most redundant platforms is the Affy array with an average 1.98 probe sets per gene.

### Gene coverage from mouse whole genome microarrays and oligonucleotide sets

**Figure 6:**
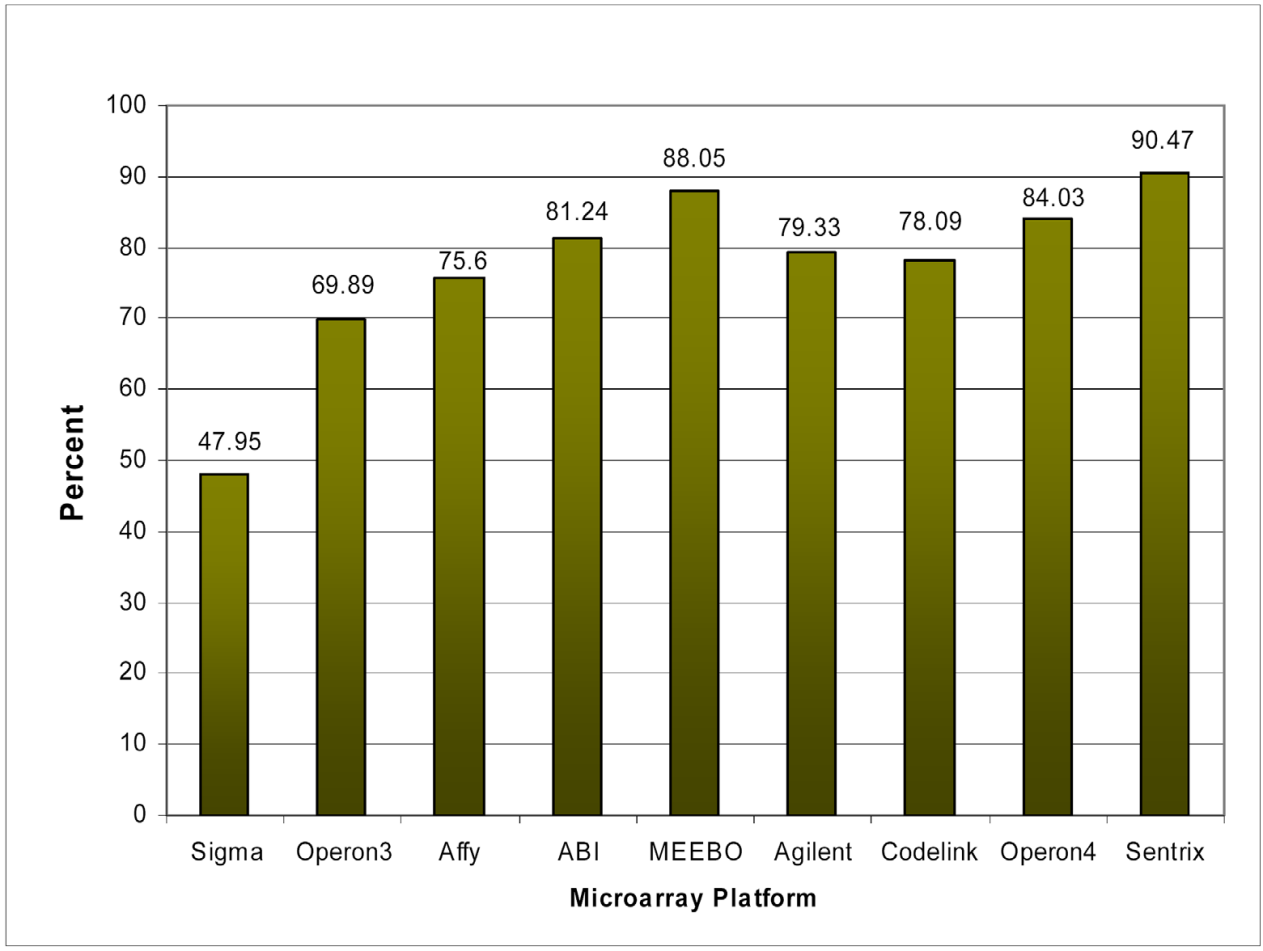
**Gene coverage comparison between mouse microarray platforms and oligonucleotide sets**. Coverage percent is calculated as (number of genes tested)/(number of genes in the genome) × 100. The total number of genes in the genome was calculated as the number of Entrez Genes that could be mapped to a unique position in the UCSC Genome Browser Database, mouse genome Build 35.1. Microarrays are ordered from left to right by date of release. Although this was not known for every platform, we estimated the date of release using the available information both from vendor web sites and from personal communications with their costumer support (details in [Additional file 1]).

Gene coverage was estimated as the proportion of Entrez Genes with a unique position in the UCSC Genome database that is tested by a given microarray platform. The genome wide coverage varied from different platforms, ranging from 47.95% to 90.47% (Table 4 and Figure 6). The lowest coverage was observed, as expected, for the oldest platform, the Sigma oligoset, with a total of 16,377 probes testing 12,188 genes. Agilent and Codelink showed very similar coverage levels (79.33% and 78.09%, respectively). Sentrix is the ready-to-use mouse microarray with the highest gene coverage, with 90.47% of the publicly available genes tested. This was followed by the public oligonucleotide set, MEEBO (88.05%), that Sentrix was based on. The Operon AROS Arrays Oligosets showed clear improvement in gene coverage levels as new releases of their oligo data set have become available. The latest release (Operon4) shows 84.03% coverage, higher than Operon3 which only covered 69.89% and even Agilent and Affy (79.33% and75.6%, respectively).

### Gene coverage by chromosome

**Figure 7:**
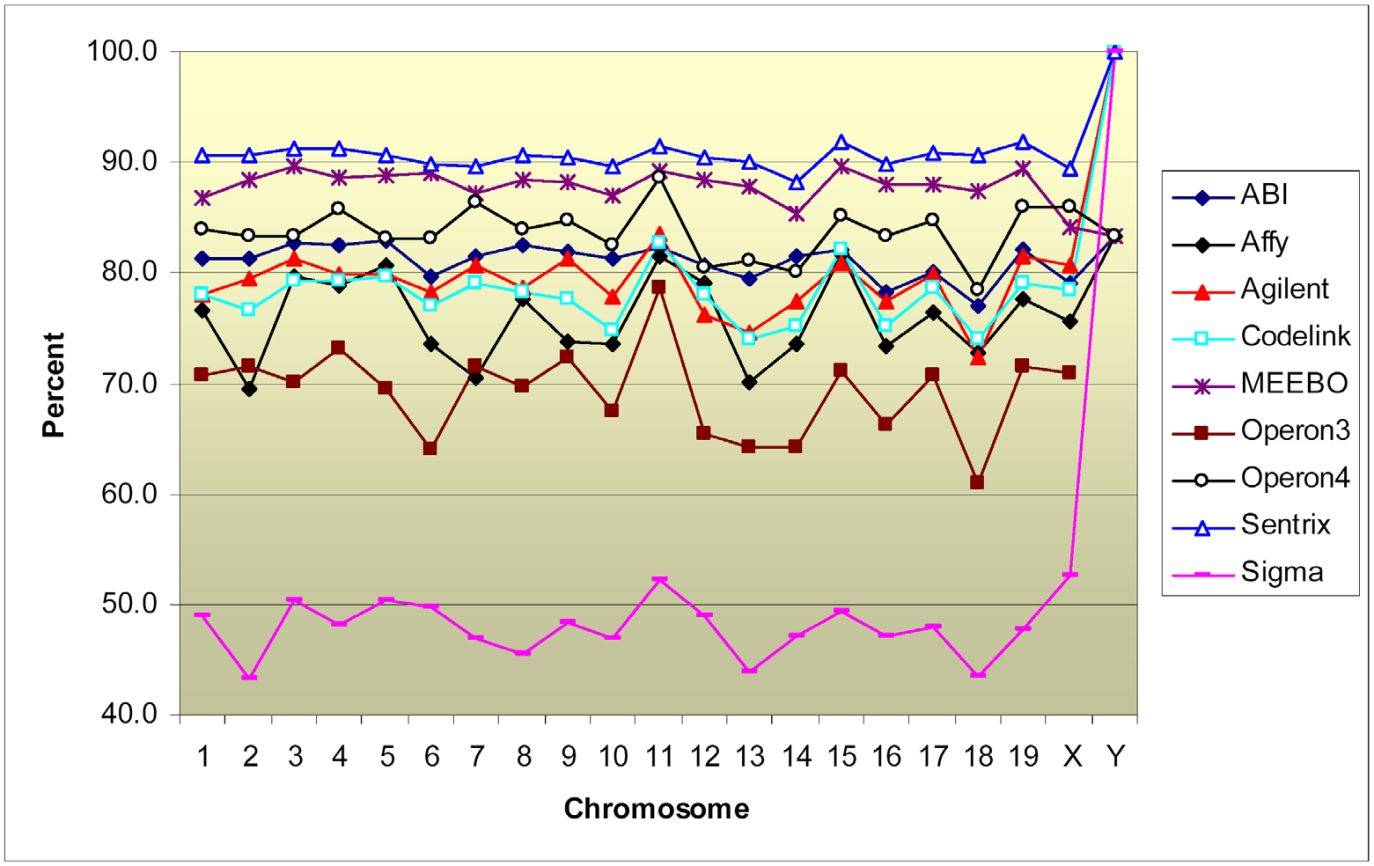
Comparative view of gene coverage (%) between microarray platforms for each mouse chromosome.

**Figure 8:**
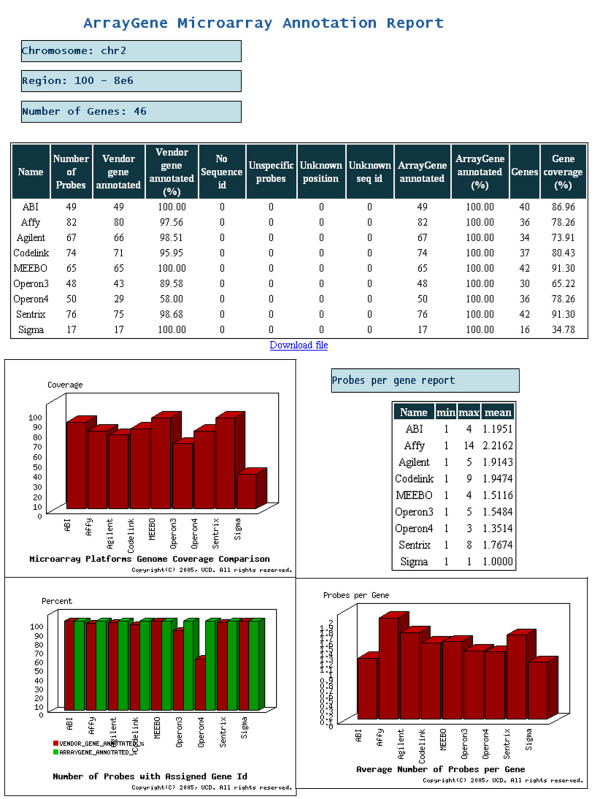
**Example of online summary report of gene annotations of microarrays platforms produced by ArrayGene**. The researcher can query the database for a comparison of gene coverage for the whole genome or for any specific region of a given chromosome. The example shows a comparison for genes in mouse chromosome 2, between 100 and 8,000,000 bp (Genome Build 35.1). The results are shown in form of tables and color bar graphs.

The differences between platforms in terms of gene coverage are well conserved across chromosomes (Figure 7). Some changes, though, can be observed in specific cases. For instance, the Affymetrix platform has a particularly low coverage for mouse chromosome 2 and 7 (69.4% and 70.5%), being outperformed even by the older Operon3 oligoset (71.6% and 71.5%, respectively). For the complete list of gene coverage by chromosome per platform see [Additional file 2] of the supplementary material. The database can be easily queried for gene coverage comparison on any region of any chromosome. Figure 8 shows an example output report for a 7.9 MB region of mouse chromosome 2.

## Discussion

The use of microarrays has recently been extended to the study of natural genetic variation affecting the expression of genes at the transcript level. Such techniques, originally coined as Genetical Genomics [15], treats gene expression as a quantitative trait suitable for QTL analysis. Using this approach, several groups have been able to detect loci affecting the expression of thousands of genes, both in cis and trans, in yeast [16,17], mouse [18], and humans [18,19]. Furthermore, Schadt *et al*. [20] were able to identify cis-acting QTL for gene expression (eQTL) causing obesity in mouse. However, the success of this approach depends heavily on the level of gene coverage from the microarray platform being used. Previous QTL mapping knowledge can provide candidates regions for cis-eQTL that researchers would like to exhaustively test with a microarray platform. This is of prime importance when microarrays are used to compare congenic lines with background strains and the only difference between individuals is a small chromosomal region [21-23]. *A priori *knowledge of the level of gene coverage in the congenic region is essential to assess the significance of the results from such studies, creating the need for chromosome and region specific gene coverage comparisons between microarray platforms. Consistent gene identifiers are needed that can be mapped to specific locations in the genome. We have created a system to perform in-house gene annotations on sequences from a number of different sources. This system is centered on our *ArrayGene *database which maintains an extensive set of cross references between sequence identifiers and Entrez Gene ids. This system was used to annotate the sequences mapped by the UCSC Genome Browser Database with gene ids and created a gene-centered database called *Aligndb*. These databases, and a set of Perl scripts and modules, provide a platform for annotating and maintaining updated gene annotations of microarrays. We have annotated and compared gene coverage from five mouse oligonucleotide microarrays and four oligosets used for microarray spotting. Only genes that could be uniquely mapped to a single position in the genome with the UCSC Genome Browser database were included in the comparison. Genome coverage was then estimated as the proportion of such genes that is tested by a given platform. None of the platforms tested provided 100% coverage of the mouse genome, and their level of coverage depended greatly on the date of release. Newer sets have better coverage, most likely due to a better state of genome assembly and annotation at the time of design. It should be noted that nonetheless the highest coverage was found in the newest commercial microarray available, Sentrix by Illumina, it was followed by a non-commercial oligoset that was the basis of the Sentrix platform, called MEEBO (Exonic Evidence Based Oligonucleotide). This oligoset was developed by a collaborative effort between researchers at UCSF, Stanford, Rockefeller, Basel, and the Stowers Institute and it was based on an early draft of the genome, NCBI Build 30 (Jan 2003), which is previous to the genomic assemblies used by most of the arrays tested here (Table S4 for details).

Significant differences were also found in the level of redundancy (Figure 5) and the amount of information that vendors provide for their platforms. The latter is a critical point at the time of data analysis if biological inferences are to be made from expression data. For instance, not all probes in the MEEBO array are annotated. The authors only provide an accession number for 81.8% of non-control probes. They do provide a gene symbol for 97% of them, but the user has no means of finding the gene from sequence-specific annotations alone for 18.2% of the non-control probes. However, the case is different for Operon4 array where although 99% of probes are annotated with at least one sequence identifier, many of them (6,715) could not be associated with any Entrez Gene, representing 78.3% of the not annotated probes by *ArrayGene *for this platform. This is also the case for 6,309 probes in the Codelink array with no Entrez Gene association (Unknown seq id probes in Figure 4). Whether this is a consequence of using obsolete accession numbers that are not included in current development of Entrez Gene or Unigene, or if it reflects big omissions in the curation of Entrez Gene, this should not affect the comparison in the present study. We expect that by imposing the same restrictions and conditions to all platforms our comparison can reflect real differences in gene coverage level. However we are aware that older platforms may be penalized because of the use of old sequences or ESTs that are not included in the Entrez Gene database and RefSeqs that are obsolete. It also must be noted that by using gene annotations such as gene symbols or Unigene identifications provided by vendors, we assume that the annotation properly associates the probe with the source sequence. This not only can add errors to the gene annotation process, but can increase the percentage of unspecific-probe calls since gene symbols sometimes can be associated with more than one gene. However, in some platforms, gene symbols were the only probe annotation available and not using them would restrict the inclusion of those platforms in the present study. Therefore, we opted for using gene symbols as probe annotations but not Unigene ids since these are automated sequence clusters with no human curation, are not stable and could lead to errors, especially overestimation of unspecific probes if the UniGene build used at the time of generation of the gene list is different from that used to build the *genexref *table of *ArrayGene*.

Although we believe that our experimental design allowed for a fair and comprehensive comparison of gene coverage between platforms, this work also led us to identifying obstacles that researchers will encounter when trying to answer the questions of how many genes are being tested in a given platform and what is its level of coverage for a given region. The problems that we have encountered to perform optimal gene annotations of probes are associated with the lack of complete gene lists with essential information for the probes. Furthermore, accession numbers may become obsolete and gene names can change with time. The only data that is completely uniform and stable is the sequence of the probe. Sequences would allow any person to perform an alignment with the genome and identify the position being tested to determine what are the genes been tested. The accession number of the original sequence from which the probe was designed can also provide valuable information for the user. Unfortunately, these are not always readily available and for this reason we could not use them in this study. For proprietary reasons vendors very often provide only a consensus or representative sequence identifier which does not necessarily correspond to the original sequences used for probe design. Other probe annotations such as RefSeq accession, gene symbol, gene ontology classification, pathway, etc, can also be very useful for any potential user of the platform. However, from this work we see the need for standardization of a minimal set of information that vendors should provide along with the microarray itself. This should include: (1) accession number or other appropriate identifier for the original sequence used to design the probe; (2) database source of the sequence; and (3) type of probe (test vs. control). The position of the probe within the source sequence or the probe sequence is also invaluable information that, for example, would allow the user to assess possible hybridization artifacts due to polymorphisms between the target RNA and probe. Additional information such as RefSeqs id, gene name, gene symbol, and genomic position of the probe can also be very useful, helping the user to avoid performing genome-wide error prone annotations. These higher order annotations can become obsolete with the release of new genomic information, and constant updates would be needed for vendors to remain current. Some microarray vendors have already taken this approach and we believe that this will provide them with a marketing advantage, even if the features and quality of the microarrays themselves are comparably similar.

## Conclusion

The results of this study provide researchers with a comparison of the level of gene coverage of different microarrays platforms for the mouse genome, which is particularly useful for genetical genomic studies. The Sentrix microarray by Illumina and the MEEBO public oligo set that this array is based on provide the highest gene coverage of the mouse genome. The bioinformatics tools generated here can prove useful for microarray users studying other organisms with a sequenced genome. The annotation tools included in the ArrayGene software are flexible enough to be easily adapted to use input files from any database that associates genes with sequence ids. We also provide comments on the limitations from current microarray annotations to encourage the scientific community to develop minimum annotation guidelines that designers and vendors of microarrays can follow to better fulfill research needs.

## Methods

### Gene definition

The Entrez Gene database at NCBI was used as the reference to assign consistent gene annotations across platforms. The Entrez Gene database groups GenBank and RefSeq accession numbers and gene symbols by unique, non redundant and traceable gene ids [8]. These ids could also be cross-referenced to ENSEMBL transcripts and genes, and to any id that can be related to a GenBank accession number. Tables associating gene ids to GenBank and RefSeq sequences were obtained from the NCBI ftp server (files downloaded on December 15^th ^2005). A third table associating UCSC Known Genes to ENSEMBL transcripts was obtained from the UCSC ftp server (used files and source URLs in Table 1). A single table called *genexref *was created in the *ArrayGene *MySQL database, with three columns: gene id (from Entrez), sequence id, and type of sequence (i.e. Ensembl, genomic, mRNA, protein, RefSeq, symbol, synonym, NIA transcript, or Unigene). This table is the core of the gene annotation process of probes in microarrays. Only current Entrez Gene ids were used. The file gene_history from NCBI was used to delete obsolete genes gene ids from the database.

### Genomic location for genes

The physical location of genes was defined as the position where any sequence associated with the gene could be aligned with confidence by sequence comparison. Genomic alignment results were obtained from the UCSC Genome Browser Database [14] for the August 2005 mouse genome assembly (Build 35.1). Text files from this server contain the results from aligning all mouse mRNA sequences deposited at GenBank against the mouse genome sequence using BLAT. All sequence alignments in the UCSC database have at least 98% identity. The track of "Known Genes" in the UCSC Genome Browser provides genomic coordinates only for mRNA that could be associated with a protein in SWISS-PROT, TrEMBL, or TrEMBL-NEW. Similarly, the track called RefSeq Gene contains codon and intron positions for RefSeq sequences. UCSC tracks were used in a hierarchical order: 1) Known Genes, 2) RefSeq Genes, and 2) mRNA sequences. Genes mapping to unordered scaffolds or to multiple positions from BLAT alignments were not considered. A table labeled *genemap *was created in MySQL to store the best, if any, mapping information for every gene following the above criteria. The table stores the genomic coordinates from a single sequence per gene. As a result of importing these tracks in hierarchical order, coordinates from known Genes are preferred over RefSeqs, and these are over mRNA ones. The *genemap *table is updated with every new release of the mouse alignment from NCBI. The table is stored in an alignment-specific database herein called *Aligndb*. The actual name of this database is provided by the user when a new genome alignment is available, and the current database is called *mm7*, in reference to the name provided by the UCSC genome browser to the annotated Build 35.1 assembly. The proportional contribution from each UCSC track to the *genemap *table is shown in Table 2. This table shows the number of genes in each track that could not be used because they mapped to multiple positions in the genome or to unordered scaffolds. The last column of the table shows the number of genes for each track that could be mapped to a unique known position in the genome and that were not already present in the database (i.e. were not included in any track previously imported).

### Database architecture and maintenance

Two MySQL databases were created and are maintained separately. The *ArrayGene *database contains the *genexref *table which provides cross references between sequence identifiers and Entrez Genes and the *arrays_table *table storing all the information about microarray annotations (Figure 1). A second database is created every time a new mouse genome build is released. This database contains the *genemap *table providing mapping positions for genes probed in microarrays. The *ArrayGene *package was created to build and maintain both databases using command line and web forms and is included as [Additional file 4] in this report. Future new versions of the software will be available from the authors' website [24]. This system is a generic tool for the comparison of gene coverage providing a user interface to generate reports and can be used for any species with a sequenced genome and a database of genes associated with sequences, which are used to produce probes in microarrays. The input of files for both gene information and gene mapping are format independent and the programs can be customized through command line options to use any data file that is in tabular form. The software is distributed along with a user manual under the General Public License V.2 [25] and is freely available for the research community. No genomic or microarray data is included in the package and the user must follow the instructions included with the software to install and populate the database. The software depends on MySQL v. 3.23.50 or later versions, Perl (only tested on v. 5.8.2) and on some Perl modules described in installation instructions of the User Guide included as [Additional file 3].

### Gene annotation of microarray probes

The *import_array *Perl script is designed to extract the probe annotations provided by vendors for their platforms in files commonly called *gene lists*. The script can parse a text file, extract probe and sequence ids (even from fasta-style description lines), connect to the *ArrayGene *database and find the Entrez Gene id for each probe. Finally, *import_array *can either write an output file with genomic annotations for probes or create a table in the database with this information. When multiple sequences are associated with a given probe in the gene list the Perl script checks if they all match the same gene. The program can look for sequence ids in 3 columns maximum, and it can detect inconsistencies between them if they point to different Entrez Gene ids in the database. It also detects single sequences that are associated with more than one gene in the Entrez Gene database. In any of these two cases, since a unique gene cannot be associated with a probe it is annotated as a cross hybridizing probe. The ids of all genes associated with that probe are stored but are not used in genome coverage comparisons. Reports about gene coverage in annotated microarrays are done by a series of CGI scripts providing an intuitive web interface to the database.

### Microarray platforms and oligonucleotide sets

This study compared the gene coverage of mouse whole genome microarray platforms that are currently available to investigators. One older oligoset was also included to evaluate coverage improvements with time of release ([Additional file 1] in supplementary material provides time of release for some platforms, URL and filename for the Genelists used here). We compared four commercial one-color arrays, Affymetrix [3] Mouse Genome 430 2.0 Array, Amersham [26] Codelink Mouse Whole Genome, Sentrix Mouse-6 Expression BeadChip from Illumina [27], and Applied Biosystems [28] Mouse Genome Survey. We also compared the two-color Agilent [29] Mouse Oligo Microarray Kit; the commercial oligoset Operon [30] Array-Ready Oligo Set V. 4 and one previous version (Operon V. 3); the Sigma-Genosys Mouse Oligonucleotide library (available through Lab on Web [31]), and the Mouse Exonic Evidence Based Oligonucleotide (MEEBO) [32] produced by a group of investigators at UCSF, Stanford, Rockefeller, Basel, and the Stowers Institute. Table 3 lists all these platforms, indicating their full and short name used throughout this paper. A special mention should be made about the ABI microarray which contains almost 4,000 proprietary sequences, which are not in the public domain. The probes in this platform were designed based on the Celera Mouse Genome Alignment (Celera, Rockville, MD), which contains gene annotations based in proprietary methods. The present study compared gene coverage by using sequence annotations equivalent to public accession numbers available for 29,195 probes. However, these probes may target genes without an exact counterpart in the public domain given large methodological differences that exist for defining a gene between the Celera and the public domain approaches.

Gene lists for every platform included in this study, except for ABI, were downloaded from the vendors' websites. ABI's gene-list was obtained directly from the vendor.

## Authors' contributions

RAV collected information from public databases and created the ArrayGene software and database and drafted the manuscript. JFM supervised the design and development of the database and guided the gene coverage comparison between microarrays platforms and helped draft the manuscript. Both authors read and approved the final manuscript.

## Supplementary Material

Additional File 1 Microarray platforms compared in this study. Table provides details of the Genelists used, including filename, URL, date of release, date updated, and date obtained. The column called annot update lists the date that the Genelist available for the platform was last updated by the vendor Click here for file

Additional File 2 Comparative gene coverage from whole mouse genome microarrays and oligo set. Table shows the number of Entrez Genes with a single genomic position in the genome by the UCSC Genome Browser Database, and the number of genes that are tested by each platform as absolute counts and as percentage from the number of genes in the chromosome. Click here for file

Additional File 4 ArrayGene software with an install script for UNIX platforms and a README file for installation and usage instructions Click here for file

Additional File 3 Reference manual for the installation and use of the ArrayGene software. The manual includes a list of system requirements and the full list of options for all the tools included in the package. Click here for file

## Figures and Tables

**Table 1:** Files used to create the local database and URLs of sources of data.

Source Name	URL	File name
NCBI		gene_info gene2accession gene2refseq gene_history gene2unigene Mm.gb_cid_lid
UCSC		knownGene refGene all_mrna knownToEnsembl

**Table 2:** Gene tracks from the UCSC Genome Browser Database used for finding genomic locations of Entrez Genes and to create the *genemap *table to store mapping information. Tracks were used hierarchically in the order shown here from top to bottom.*Genes in track *are the number of distinct Entrez genes in the Sequence Track. Genes in Multiple *positions *cannot be mapped to a unique position. Genes in *Random scaffolds *map to unordered scaffolds. Used Genes refers to the number of non redundant genes with a unique position in the genome that were imported to the *genemap *table.

Browser Track	Sequences in Track	Genes			
		
		Genes in Track	Multiple positions	Random Scaffolds	Used Genes
Known Genes	31,449	17,565	882	53	16,630
RefSeq Genes	18,863	17,531	292	104	2,306
mRNA	219,260	24,850	1,107	116	6,480
**Total**	**269,572**				**25,416**

**Table 3:** Mouse oligonucleotide microarray and oligoset platforms included in this study.

Vendor	Product	Part Number	Short Name
Affymetrix	Mouse Genome 430 2.0 Array	900497	Affy
Agilent	Mouse Oligo Microarray Kit	G4122A	Agilent
Amersham	Codelink Mouse Whole Genome	300033	Codelink
Illumina	Sentrix^® ^Mouse-6 Expression BeadChip	Mouse-6	Sentrix
Invitrogen	MEEBO mouse genome set	OL-10-122	MEEBO
LabOnWeb	Mouse Oligonucleotide Library	MOULIB96T	Sigma
Operon	Array-Ready Oligo Set V.4	810619	Operon4
Operon	Array-Ready Oligo Set V.3	NA	Operon3
ABI	Mouse Genome Survey	4345064	ABI

**Table 4:** Summary of gene probe annotations for whole genome mouse microarrays and oligosets. The vendor annotated percent represents the fraction of probes that have a gene id provided by the vendor.*ArrayGene *annotations are automatically performed by the *ArrayGene *software by associating an Entrez Gene id to the sequence ids in gene lists provided by vendors. Gene coverage represents the percentage of uniquely mapped genes in the genome that are tested by gene-specific probes in the microarrays and oligosets.

Short Name	Number of Probes	Vendor annotated	Vendor annotated (%)	ArrayGene annotated	ArrayGene annotated (%)	Genes	Gene coverage
Sigma	16,377	11,785	72.0	13,119	80.1	12,188	47.95
Operon3	32,149	26,245	81.6	23,298	72.5	17,764	69.89
Affy	45,102	40,627	90.1	38,086	84.4	19,214	75.6
ABI	33,012*	33,012	100.0	24,166	73.2	20,647	81.24
MEEBO	38,524	35,058	91.0	33,164	86.1	22,378	88.05
Agilent	41,174	34,866	84.7	33,709	81.9	20,163	79.33
Codelink	37,797	30,659	81.1	29,245	77.4	19,848	78.09
Operon4	36,232	18,102	50.0	27,659	76.3	21,357	84.03
Sentrix	46,133	44,399	96.2	37,895	82.1	22,993	90.47

* *Includes 3,817 probes targeting Celera genes with no public homolog. These probes are not annotated by ArrayGene and do not contribute the estimate of gene coverage*
