# Supplementary material for: Comparison of gene coverage of mouse oligonucleotide microarray platforms
Source: BMC Genomics. 2006 Mar 21;7:58. doi: 10.1186/1471-2164-7-58 (PMC1440853; doi:10.1186/1471-2164-7-58)
Supplement: Additional File 1 — Microarray platforms compared in this study. Table provides details of the Genelists used, including filename, URL, date of release, date updated, and date obtained. The column called annot update lists the date that the Genelist available for the platform was last updated by the vendor [file 1471-2164-7-58-S1.doc]

| **Table S1.** Microarray platforms compared in this study. The second part of the table provides the URLs of the probe annotation files used for the comparison. | | | | | | | | | | | | | | | | | | | |
| --- | --- | --- | --- | --- | --- | --- | --- | --- | --- | --- | --- | --- | --- | --- | --- | --- | --- | --- | --- |
| Platform | | Vendor | | Vendor URL | Product | | Part Number | | Date Released | Genome alignment | | | Date alignment | | Annot update | | | | Date downloaded |
| 1 | | Affymetrix | | www.affymetrix.com | Mouse Genome 430 2.0 Array | | 900497 | | 03/01/2004 | MGSC | | | Apr-02 | | 09/22/2005 | | | | 11/24/2005 |
| 2 | | Agilent | | www.agilent.com | Mouse Oligo Microarray Kit | | G4122A | | 10/27/2004 | Build 32 | | | Jan-04 | | 10/10/2005 | | | | 12/12/2005 |
| 3 | | Amersham | | www.amershambiosciences.com | Codelink Mouse Whole Genome | | 300033 | | NA | Build 33 | | | Sep-04 | | 07/11/2005 | | | | 12/12/2005 |
| 4 | | Illumina | | www.illumina.com | Sentrix® Mouse-6 Expression BeadChip | | Mouse-6 | | 2005 | Build 30^†^ | | | Jan-03 | | 08/31/2005 | | | | 12/12/2005 |
| 5 | | Invitrogen | | arrays.ucsf.edu/ | MEEBO mouse genome set | | OL-10-122 | | June 2004* | Build 30 | | | Jan-03 | | 05/17/2005 | | | | 12/12/2005 |
| 6 | | LabOnWeb | | www.labonweb.com | Mouse Oligonucleotide Library | | MOULIB96T | | 2002 | NA | | | NA | | 01/03/2003 | | | | 12/12/2005 |
| 7 | | Operon | | www.operon.com | Array-Ready Oligo Set V.4 | | 810619 | | NA | Build 33 | | | Sep-04 | | 03/01/2004 | | | | 12/12/2005 |
| 8 | | Operon | | www.operon.com | Array-Ready Oligo Set V.3 | | NA | | NA | Build 30 | | | Jan-03 | | 03/01/2004 | | | | 12/12/2005 |
| 9 | | ABI | | www.appliedbiosystems.com | Mouse Genome Survey | | 4345064 | | NA | Celera | | | NA | | NA | | | | 05/16/2005 |
| * *represents the date of release of the oligoset by the Stanford Microarray Core Facility (http://www.microarray.org/sfgf/jsp/home.jsp)* | | | | | | | | | | | | | | | | | | | |
| ^†^ *Sentrix was mainly based on MEEBO and then on Build 30 but probes were added on 2005* | | | | | | | |  | | |  |  |  | |  | | | |  |
|  |  | |  | | |  | |  | | |  |  |  | |  | | | |  |
| **Table S1. *cont.*** | | | | | | | | | | | | | | | | | | |  |
| Platform | | Filename | | | | Product URL | |  | | |  | |  |  | |  | |  | |
| 1 | | Mouse430_2_annot.csv | | | | http://www.affymetrix.com/products/arrays/specific/mouse430_2.affx | | | | | | |  |  | |  | |  | |
| 2 | | 012694_D_AA_20051010.txt | | | | http://www.chem.agilent.com/Scripts/PDS.asp?lPage=17228 | | | | | | |  |  | |  | |  | |
| 3 | | 300033.txt | | | | http://www6.amershambiosciences.com/aptrix/upp01077.nsf/Content/codelink_gene_lists | | | | | | | | | |  | |  | |
| 4 | | Mouse6_Gene_List.txt | | | | http://www.illumina.com/General/products/arraysreagents/Excel/Mouse6_Gene_List.xls | | | | | | | | | |  | |  | |
| 5 | | MEEBO_Annotations_051705.txt | | | | http://alizadehlab.stanford.edu/%7Eash/meebochip/specifications/llids/MEEBO_Annotations_051705.txt | | | | | | | | | | |  | | |
| 6 | | MOULIB96T_ps.xls | | | | http://www.labonweb.com/cgi-bin/chips/public_loader.cgi?file=MOULIB96T_ps.xls | | | | | | | |  | |  | |  | |
| 7 | | mouse_V4.0.1_genelist_s+.xls | | | | http://omad.operon.com/download/storage/mouse_V4.0.1_genelist_s+.xls.zip | | | | | | | |  | |  | |  | |
| 8 | | mouse_upgrade_V2.0.3_genelist_s+.xls | | | | http://omad.operon.com/download/storage/mouse_V2.0.3_genelist_s+.xls.zip | | | | | | | |  | |  | |  | |
| 9 | | ABI mouse_geo_equivalent_file.txt | | | | File directly obtained from Applied Biosystems by personla communication | | | | | | |  |  | |  | |  | |
