# Supplementary material for: Comparison of gene coverage of mouse oligonucleotide microarray platforms
Source: BMC Genomics. 2006 Mar 21;7:58. doi: 10.1186/1471-2164-7-58 (PMC1440853; doi:10.1186/1471-2164-7-58)
Supplement: Additional File 2 — Comparative gene coverage from whole mouse genome microarrays and oligo set. Table shows the number of Entrez Genes with a single genomic position in the genome by the UCSC Genome Browser Database, and the number of genes that are tested by each platform as absolute counts and as percentage from the number of genes in the chromosome. [file 1471-2164-7-58-S2.doc]

| **Table S2.** Comparative gene coverage from whole mouse genome microarrays and oligo set. The second column shows the number of Entrez Genes with a single genomic position in the genome by the UCSC Genome Browser Database. The following columns present the number of genes that are tested by each platform as absolute counts and as percentage from the number of genes in the chromosome. | | | | | | | | | | |
| --- | --- | --- | --- | --- | --- | --- | --- | --- | --- | --- |
| Chr | Entrez Genes | ABI (%) | Affy (%) | Agilent (%) | Codelink (%) | MEEBO (%) | Operon3 (%) | Operon4 (%) | Sentrix (%) | Sigma (%) |
| **1** | 1,491 | 1,213 (81.4) | 1,141 (76.5) | 1,163 (78.0) | 1,165 (78.1) | 1,293 (86.7) | 1,053 (70.6) | 1,251 (83.9) | 1,351 (90.6) | 729 (48.9) |
| **2** | 2,161 | 1,757 (81.3) | 1,500 (69.4) | 1,717 (79.5) | 1,657 (76.7) | 1,909 (88.3) | 1,547 (71.6) | 1,801 (83.3) | 1,957 (90.6) | 936 (43.3) |
| **3** | 1,245 | 1,030 (82.7) | 991 (79.6) | 1,011 (81.2) | 987 (79.3) | 1,116 (89.6) | 874 (70.2) | 1,037 (83.3) | 1,135 (91.2) | 626 (50.3) |
| **4** | 1,452 | 1,197 (82.4) | 1,146 (78.9) | 1,159 (79.8) | 1,150 (79.2) | 1,286 (88.6) | 1,063 (73.2) | 1,245 (85.7) | 1,326 (91.3) | 699 (48.1) |
| **5** | 1,510 | 1,253 (83.0) | 1,217 (80.6) | 1,205 (79.8) | 1,203 (79.7) | 1,341 (88.8) | 1,050 (69.5) | 1,255 (83.1) | 1,368 (90.6) | 762 (50.5) |
| **6** | 1,435 | 1,144 (79.7) | 1,056 (73.6) | 1,123 (78.3) | 1,104 (76.9) | 1,278 (89.1) | 917 (63.9) | 1,192 (83.1) | 1,290 (89.9) | 713 (49.7) |
| **7** | 2,066 | 1,683 (81.5) | 1,456 (70.5) | 1,667 (80.7) | 1,633 (79.0) | 1,803 (87.3) | 1,478 (71.5) | 1,785 (86.4) | 1,851 (89.6) | 968 (46.9) |
| **8** | 1,275 | 1,053 (82.6) | 989 (77.6) | 1,002 (78.6) | 997 (78.2) | 1,128 (88.5) | 888 (69.6) | 1,071 (84.0) | 1,157 (90.7) | 580 (45.5) |
| **9** | 1,434 | 1,174 (81.9) | 1,057 (73.7) | 1,166 (81.3) | 1,113 (77.6) | 1,264 (88.1) | 1,038 (72.4) | 1,215 (84.7) | 1,296 (90.4) | 692 (48.3) |
| **10** | 1,250 | 1,016 (81.3) | 919 (73.5) | 972 (77.8) | 936 (74.9) | 1,087 (87.0) | 843 (67.4) | 1,031 (82.5) | 1,121 (89.7) | 586 (46.9) |
| **11** | 1,871 | 1,540 (82.3) | 1,525 (81.5) | 1,563 (83.5) | 1,546 (82.6) | 1,671 (89.3) | 1,472 (78.7) | 1,657 (88.6) | 1,712 (91.5) | 975 (52.1) |
| **12** | 860 | 693 (80.6) | 680 (79.1) | 655 (76.2) | 671 (78.0) | 760 (88.4) | 562 (65.3) | 692 (80.5) | 777 (90.3) | 421 (49.0) |
| **13** | 1,021 | 811 (79.4) | 716 (70.1) | 762 (74.6) | 755 (73.9) | 897 (87.9) | 655 (64.2) | 827 (81.0) | 920 (90.1) | 447 (43.8) |
| **14** | 966 | 788 (81.6) | 710 (73.5) | 747 (77.3) | 727 (75.3) | 824 (85.3) | 620 (64.2) | 774 (80.1) | 853 (88.3) | 456 (47.2) |
| **15** | 958 | 786 (82.0) | 783 (81.7) | 775 (80.9) | 786 (82.0) | 859 (89.7) | 681 (71.1) | 815 (85.1) | 880 (91.9) | 473 (49.4) |
| **16** | 860 | 672 (78.1) | 631 (73.4) | 665 (77.3) | 647 (75.2) | 757 (88.0) | 570 (66.3) | 716 (83.3) | 772 (89.8) | 405 (47.1) |
| **17** | 1,168 | 935 (80.1) | 892 (76.4) | 932 (79.8) | 918 (78.6) | 1,028 (88.0) | 825 (70.6) | 991 (84.8) | 1,060 (90.8) | 561 (48.0) |
| **18** | 688 | 530 (77.0) | 500 (72.7) | 498 (72.4) | 509 (74.0) | 601 (87.4) | 420 (61.0) | 539 (78.3) | 624 (90.7) | 299 (43.5) |
| **19** | 828 | 680 (82.1) | 643 (77.7) | 674 (81.4) | 655 (79.1) | 740 (89.4) | 592 (71.5) | 711 (85.9) | 760 (91.8) | 396 (47.8) |
| **X** | 871 | 688 (79.0) | 658 (75.5) | 702 (80.6) | 684 (78.5) | 732 (84.0) | 617 (70.8) | 748 (85.9) | 778 (89.3) | 459 (52.7) |
| **Y** | 6 | 5 (83.3) | 5 (83.3) | 6 (100.0) | 6 (100.0) | 5 (83.3) | 0 (0.0) | 5 (83.3) | 6 (100.0) | 6 (100.0) |
