# Supplementary material for: Comparison of gene coverage of mouse oligonucleotide microarray platforms
Source: BMC Genomics. 2006 Mar 21;7:58. doi: 10.1186/1471-2164-7-58 (PMC1440853; doi:10.1186/1471-2164-7-58)
Supplement: Additional File 4 — ArrayGene software with an install script for UNIX platforms and a README file for installation and usage instructions [file 1471-2164-7-58-S4.zip › ArrayGene-0.2/pub/html/add_vendor.html]

ArrayGene: Add New Vendor


# Enter Information for new vendor:

|  |  |
| --- | --- |
| Vendor Name  | |
| URL  | |
| Admin Password  | |
