# Supplementary material for: Comparison of gene coverage of mouse oligonucleotide microarray platforms
Source: BMC Genomics. 2006 Mar 21;7:58. doi: 10.1186/1471-2164-7-58 (PMC1440853; doi:10.1186/1471-2164-7-58)
Supplement: Additional File 4 — ArrayGene software with an install script for UNIX platforms and a README file for installation and usage instructions [file 1471-2164-7-58-S4.zip › ArrayGene-0.2/pub/html/list_genes_example.htm]

xml version="1.0" encoding="iso-8859-1"?

ArrayGene Microarray Annotation Report

# ArrayGene: List of Genes

## Chromosome: all

## Region: -

### Number of Genes Tested by Chromosome per Platform

| Chromosome | Genes | MEEBO | Agilent | Sigma | Operon3 | ABI | Operon2 | Amersham | Operon4 | Affy |
| --- | --- | --- | --- | --- | --- | --- | --- | --- | --- | --- |
| chrX | 828 | 751 | 697 | 566 | 584 | 686 | 487 | 652 | 720 | 643 |
| chrY | 9 | 8 | 8 | 7 | 0 | 7 | 9 | 9 | 8 | 8 |
| chr1 | 1462 | 1314 | 1152 | 969 | 1016 | 1202 | 847 | 1124 | 1214 | 1096 |
| chr2 | 2129 | 1924 | 1687 | 1251 | 1501 | 1728 | 1113 | 1610 | 1758 | 1457 |
| chr3 | 1216 | 1126 | 985 | 831 | 855 | 1025 | 763 | 944 | 1020 | 952 |
| chr4 | 1439 | 1311 | 1163 | 971 | 1034 | 1188 | 831 | 1117 | 1227 | 1124 |
| chr5 | 1475 | 1339 | 1186 | 1016 | 1012 | 1230 | 900 | 1153 | 1211 | 1159 |
| chr6 | 1397 | 1270 | 1088 | 915 | 896 | 1138 | 782 | 1054 | 1167 | 1020 |
| chr7 | 1982 | 1779 | 1578 | 1204 | 1434 | 1643 | 1045 | 1556 | 1712 | 1372 |
| chr8 | 1231 | 1124 | 980 | 801 | 849 | 1029 | 718 | 951 | 1034 | 943 |
| chr9 | 1416 | 1275 | 1146 | 921 | 1012 | 1162 | 798 | 1069 | 1191 | 1019 |
| chr10 | 1221 | 1096 | 940 | 792 | 808 | 1002 | 685 | 902 | 995 | 884 |
| chr11 | 1865 | 1697 | 1532 | 1234 | 1417 | 1531 | 1145 | 1471 | 1625 | 1467 |
| chr12 | 832 | 766 | 637 | 582 | 544 | 686 | 497 | 623 | 675 | 642 |
| chr13 | 999 | 898 | 740 | 613 | 626 | 794 | 523 | 726 | 804 | 679 |
| chr14 | 926 | 830 | 725 | 607 | 590 | 762 | 528 | 688 | 749 | 679 |
| chr15 | 947 | 866 | 768 | 635 | 666 | 782 | 594 | 774 | 802 | 760 |
| chr16 | 823 | 742 | 640 | 543 | 546 | 652 | 482 | 608 | 685 | 592 |
| chr17 | 1117 | 1020 | 890 | 716 | 791 | 917 | 667 | 883 | 953 | 844 |
| chr18 | 671 | 610 | 490 | 413 | 400 | 529 | 397 | 481 | 523 | 478 |
| chr19 | 835 | 770 | 685 | 550 | 593 | 691 | 515 | 658 | 714 | 629 |

Download file

### Gene Coverage by Chromosome per Platform

| Chromosome | MEEBO | Agilent | Sigma | Operon3 | ABI | Operon2 | Amersham | Operon4 | Affy |
| --- | --- | --- | --- | --- | --- | --- | --- | --- | --- |
| chrX | 90.7 | 84.2 | 68.4 | 70.5 | 82.9 | 58.8 | 78.7 | 87.0 | 77.7 |
| chrY | 88.9 | 88.9 | 77.8 | 0.0 | 77.8 | 100.0 | 100.0 | 88.9 | 88.9 |
| chr1 | 89.9 | 78.8 | 66.3 | 69.5 | 82.2 | 57.9 | 76.9 | 83.0 | 75.0 |
| chr2 | 90.4 | 79.2 | 58.8 | 70.5 | 81.2 | 52.3 | 75.6 | 82.6 | 68.4 |
| chr3 | 92.6 | 81.0 | 68.3 | 70.3 | 84.3 | 62.7 | 77.6 | 83.9 | 78.3 |
| chr4 | 91.1 | 80.8 | 67.5 | 71.9 | 82.6 | 57.7 | 77.6 | 85.3 | 78.1 |
| chr5 | 90.8 | 80.4 | 68.9 | 68.6 | 83.4 | 61.0 | 78.2 | 82.1 | 78.6 |
| chr6 | 90.9 | 77.9 | 65.5 | 64.1 | 81.5 | 56.0 | 75.4 | 83.5 | 73.0 |
| chr7 | 89.8 | 79.6 | 60.7 | 72.4 | 82.9 | 52.7 | 78.5 | 86.4 | 69.2 |
| chr8 | 91.3 | 79.6 | 65.1 | 69.0 | 83.6 | 58.3 | 77.3 | 84.0 | 76.6 |
| chr9 | 90.0 | 80.9 | 65.0 | 71.5 | 82.1 | 56.4 | 75.5 | 84.1 | 72.0 |
| chr10 | 89.8 | 77.0 | 64.9 | 66.2 | 82.1 | 56.1 | 73.9 | 81.5 | 72.4 |
| chr11 | 91.0 | 82.1 | 66.2 | 76.0 | 82.1 | 61.4 | 78.9 | 87.1 | 78.7 |
| chr12 | 92.1 | 76.6 | 70.0 | 65.4 | 82.5 | 59.7 | 74.9 | 81.1 | 77.2 |
| chr13 | 89.9 | 74.1 | 61.4 | 62.7 | 79.5 | 52.4 | 72.7 | 80.5 | 68.0 |
| chr14 | 89.6 | 78.3 | 65.6 | 63.7 | 82.3 | 57.0 | 74.3 | 80.9 | 73.3 |
| chr15 | 91.4 | 81.1 | 67.1 | 70.3 | 82.6 | 62.7 | 81.7 | 84.7 | 80.3 |
| chr16 | 90.2 | 77.8 | 66.0 | 66.3 | 79.2 | 58.6 | 73.9 | 83.2 | 71.9 |
| chr17 | 91.3 | 79.7 | 64.1 | 70.8 | 82.1 | 59.7 | 79.1 | 85.3 | 75.6 |
| chr18 | 90.9 | 73.0 | 61.5 | 59.6 | 78.8 | 59.2 | 71.7 | 77.9 | 71.2 |
| chr19 | 92.2 | 82.0 | 65.9 | 71.0 | 82.8 | 61.7 | 78.8 | 85.5 | 75.3 |

Download file
