# Supplementary material for: Comparison of gene coverage of mouse oligonucleotide microarray platforms
Source: BMC Genomics. 2006 Mar 21;7:58. doi: 10.1186/1471-2164-7-58 (PMC1440853; doi:10.1186/1471-2164-7-58)
Supplement: Additional File 4 — ArrayGene software with an install script for UNIX platforms and a README file for installation and usage instructions [file 1471-2164-7-58-S4.zip › ArrayGene-0.2/pub/html/main_menu.html]

ArrayGene: Main Menu


# Main Menu

- Query the ArrayGene database for gene coverage - Administration Menu (Need Password)
