# Supplementary material for: Comparison of gene coverage of mouse oligonucleotide microarray platforms
Source: BMC Genomics. 2006 Mar 21;7:58. doi: 10.1186/1471-2164-7-58 (PMC1440853; doi:10.1186/1471-2164-7-58)
Supplement: Additional File 3 — Reference manual for the installation and use of the ArrayGene software. The manual includes a list of system requirements and the full list of options for all the tools included in the package. [file 1471-2164-7-58-S3.pdf]

ArrayGene v. 0.2

User Guide

Integrated platform for storing, managing and querying genome centered microarray annotation information

Ricardo Verdugo
Dept. of Animal Science
University of California Davis
One Shields Ave
Davis, CA 95616
(raverdugo@ucdavis.edu)

Davis, California January 5, 2006

ArrayGene 0.2 beta by Ricardo Verdugo

Copyright (c) 2005 University of California Davis.

Permission is granted to anyone to make or distribute verbatim copies
of this document as received, in any medium, provided that the
copyright notice and permission notice are preserved,
and that the distributor grants the recipient permission
for further redistribution as permitted by this notice.

TABLE OF CONTENTS

TABLE OF CONTENTS.. 3
User Manual.. 4
Introduction.. 4
Portability.. 4
Target User... 4
Citing ArrayGene... 4
Bugs reports 5
Prerequisites.. 5
Install perl libraries 5
Configuration options 5
Database architecture... 6
Populating the database.. 7
ArrayGene database:... 7
Assembly database:... 8
Gene Lists files: 9
Tools in the ArrayGene Package 10
Reference of programs.. 10
import_array... 10
import_genexref.. 12
import_genemap 13
accession2gene.. 14
import_geneinfo... 15
probe2genemap... 16

User Manual

Introduction

ArrayGene is a software package that allows to creating and maintaining a database of annotations for microarray probes and to make comparisons of the level of gene coverage for any region in the genome between microarray platforms. ArrayGene provides tools thorough a command line interface to create and maintain the database, and a web interface for gene coverage queries. Some simple administrative task can also be done through dynamic web pages and more will be available in future releases.

Array gene creates reports of gene coverage, number of probes per gene, and efficiency of gene identification. Gene coverage is also provided for every chromosome if no specific genomic region is indicated.

Portability

This software has only been tested in Unix platforms (Suse 9.0 and Mac OS X Server 10.3.3). It should be possible to port it to any operative system where Perl can be run but bugs may have to be dealt with in the process.

What do I need to use Array Gene?
In order to use ArrayGene you will need:

1. Perl (and all the its modules described below)
2. A web Server
3. A MySQL server
4. Microarray GeneLists
5. Access to files associating sequence identifiers used in GeneLists and Genes
6. Access to files with genomic coordinated or genes or sequences.

Target User

Potential users of ArrayGene are biologist interested in microarray platforms for a given organism. The amount of data that is stored in the database requires large storage space (mainly for annotation source files) and RAM (2GB recommended) more commonly available in servers. Set up and maintenance must be done by experienced bioinformaticians or IT staff given the need of a running a MySQL server, a Unix operative system and the installation of all the required Perl modules and their dependencies.

Citing ArrayGene

This beta version of ArrayGene is provided as supplementary material for an article in BMC Genomics. Please cite this article when ArrayGene has been used.

Bugs reports

Please report any bugs in this software to Ricardo Verdugo at raverdugo@ucdavis.edu. I will do my best to provide support.

Prerequisites

MySQL: only tested with v 4.0 but should work with previous versions too.
 ArrayGene uses the build foreign key check capabilities for INNODB
 tables. Version 3.23.50 or higher is required.

Perl Modules (NOTE: all this modules can be obtained from CPAN. Type "man cpan" for details):

1) ExtUtils::MakeMaker
2) DBI
3) CGI
4) Getopt::Long
5) Term::ProgressBar
6) DBD::Chart

Install perl libraries

In ortehr to run ArrayGene you need to install the libraries that come with the program. Go to the library folder fomr the package root folder. Execute the commands:

```
$  perl Makefile.PL
$  make
$  sudo make install
```

Configuration options

You need to provide the directories where html and cgi scripts can be saved. Make sure that you have writing rights here and that the web client (nobody in linux and www in MacOS X by default) has read atributes.

At the top level directory of the ArrayGene distribution run:

```
$ ./configure.pl
```

After answering some questions, a configuration file is created in the /pub/conf/ directory called general.conf
Check this file to confirm the values are correct. Then run

```
$ sudo ./install.pl
```

This program will install the html and cgi files in the directories indicated to the configure.pl program. It will also create the necessary MySQL databases. You will need a MySQL root password! for this step.

Database architecture

The ArrayGene system uses a MySQL database to store all the information about genes and microarrays. A second database stores the mapping information for the genes. This allows for efficient retrieval of information through SQL queries. The database follows the following architecture:

```
------------------------------------------
| ArrayGene
|     |-genexref
|           |-gene_id-------------------|
|           |-seq_id                    |
|           |-type                      |
|                                       |
|     |-gene_info                       |
|           |-gene_id-------------------|
|           |-Symbol                    |
|           |-LocusTag                  |
|           |-Synonyms                  |
|           |-dbXrefs                   |
|           |-chromosome                |
|           |-map_location              |
|           |-description               |
|           |-type                      |
|           |-nomenclature_auth         |
|           |-nomenclature_auth_full    |
|           |-nomenclature_status       |
|                                       |
|     |-vendors                         |
|           |-id-------------|          |
|           |-Vendor         |          |
|           |-URL            |          |
|                            |          |
|     |-platforms            |          |
|           |-Id-------------|--|       |
|           |-Vendor_FK------|  |       |
|           |-Product           |       |
|           |-PN                |       |
|           |-SName             |       |
|                               |       |
|     |-arrays_table            |       |
```

```
|           |-probe_id          |       |
|           |-platform_FK-------|       |
|           |-array_name                |
|           |-probe_name                |
|           |-source_id                 |
|           |-source                    |
|           |-type                      |
|           |-refseq                    |
|           |-other_id                  |
|           |-vendor_gene               |
|           |-gene_id-------------------|
|           |                           |
----------------------------------------|----
                                        |
                                        |
      ----------------------------------|----
      | Alignmet_db                     |
      |     |-genemap                   |
      |             |-gene_id-----------|
      |             |-name
      |             |-chrom
      |             |-strand
      |             |-txStart
      |             |-txEnd
      |             |-source
      |
      ----------------------------------------
```

Populating the database

ArrayGene database:

The table genexref must be populated with cross-references between gene ids and sequences ids. A good source of data is the GeneEntrez database. Files can be downloaded from the NCBI FTP server. Useful files are:

- gene2accession
- gene_history
- gene2refseq
- gene_infoSee

See the file ftp://ftp.ncbi.nlm.nih.gov/gene/README for more information. The following file from the UCSC Genome Browser database is also useful:

- knownToEnsembl.txt

The program import_genexref located in the bin folder of the ArrayGene distribution can take flat files and populate the database. Call the program with the --help attribute to see the available options. A full set of examples of usage to populate

the database with files above follows (lines are broken for display but should be entered as single lines in the command line) :

```
$ import_genexref --input gene2refseq --gene_id 2 --xref 4
        --type refseq --filter 1 --fvalue 10090 --missing="-"

$ import_genexref --input data/NCBI/gene2refseq --gene_id 2 --xref 6
        --type refseq --filter 1 --fvalue 10090 --missing="-"

$ import_genexref --input gene2refseq --gene_id 2 --xref 8
        --type refseq --filter 1 --fvalue 10090 --missing="-"

$ import_genexref --input gene2accession --gene_id 2 --xref 4
        --type mRNA --filter 1 --fvalue 10090 --missing="-"

$ import_genexref --input gene2accession --gene_id 2 --xref 6
        --type protein --filter 1 --fvalue 10090 --missing="-"

$ import_genexref --input gene_info --gene_id 2 --xref 5
        --type synonym --filter 1 --fvalue 10090 --missing="-"
        --recsep="|"
```

You can also use files that do not directly associate a sequence ids with an Entrez Gene ids, but instead with some other id that can in turn be associated with an Entrez Gene id. This is fairly simple to do by annotating the file first with the program accessio2gene (see below) and then importing the annotated file with import_array. In this way, one can create associations between sequence ids and Entrez Gene ids. For example, the files called knownToEnsembl.txt from UCSC (ftp://hgdownload.cse.ucsc.edu/goldenPath/mm7/database) provides links between Ensembl transcript ids and accession numbers of sequences in the KnownGene track of the UCSC Genome Browser. In order to include links between Ensembl transcripts and Entrez Genes in the database, one needs first to identify the gene by using the accession number provided in the file:

```
$ accession2gene --input knownToEnsembl.txt --accession 1 --outname EnsemblToEntrez.txt
```

Now you can import the new annotated file to the database:

```
$ import_genexref --input EnsemblToEntrez --gene_id 3 --xref 2 --type ensembl --missing=""
```

Assembly database:

The files available at the UCSC Genome Browser server are useful to populate the

genemap table, but others can be used as well.

To populate the genome assembly database call the import_genemap.pl with genome annotation files as input such as those generated by UCSC. Before these files can be used, the genes associated with the mapped sequenced should be identified. This can be done with the accession2gene program that is included in the package (bin folder):

```
$ accession2gene --input knownGene.txt --accession 1 --output knownGene.txt.out

$ import_genemap.pl --input knownGene.txt.out --align mm6_2 --gene_id 13 --name 1
        --chr 2 --strand  --txstart 4 --txend 5 --password SP#6fG
```

Gene Lists files:

The import_array program is used to read and annotate probes in a gene list. The results are stored in a MySQL database. It is important to have an in depth knowledge of the nature of the file, since they change from vendor to vendor. Especially critical is to know what character is used to separate columns, and multiple records within a cell. For example, Affymetrix uses "///" to separate multiple records within a cell. Others use commas, semicolon, etc. The default "\t" for the --delim option takes cares of columns separated by tabulations. It is also important to know what is used to indicate missing records or empty cells (e.g. "---" for Affy). If missing records are indicated just by to continuous delimiters (i.e. no character) the --miss is disregarded (i.e. don't worry about it).

An example call for import_array:

```
$ import_array.pl --input MEEBO_Annotations_051705.txt --probe 2 --accession 3
        --other 5 --vgene 1 ignore 1 --recsep=" "
```

The accompanying program accession2gene can also be used to annotated a Genelist file but it does not import the results in the database. Instead, it only creates an annotated output file. This can also be done by providing an output name to import_array.

Tools in the ArrayGene Package

Reference manual of the annotation and administration tools in ArrayGene using a command line interface. The syntax to call these programs follows standard Unix rules. The arguments for the *delimiter* option in these programs is a Perl regular expression (e.g. \t is evaluated as a tabulation). The defaults for the options will depend on the values you provides in the configuration step.

An attempt has been made to make these programs portable, however they have only been tested in Unix platforms and it would be safer if users make sure that all the input files are in Unix format (i.e. having a single new line character at the end of each line). The utility dos2unix can prove useful to format the files properly. An online version of this Unix program can be found at http://www.iconv.com/dos2unix.htm.

Once ArrayGene is installed, the full list of options for any tool can be obtained by calling the program with no arguments or with the option --help.

Reference of programs

import_array

usage: import_array [-options] -i <input filename>

This program will parse a column text file, extract probe annotations such as accession numbers, RefSeq ids, Ensembl transcripts, gene symbols among other and it will do its best to find the target gene for that probe by accessing a MySQL database. Finally, it will create an annotated table in the database. This program is used to annotate and import microarray genelists into the ArrayGene system.

options
```
 -i  --input       name of input file
     --array       numeric id of the array (previously assigned by ArrayGene)
              (optional)
     --ignore      number of headers lines to ignore (default '0')
     --probes      column number (starting from 1) containing the probes ids
 -a  --accession column number (starting from 1) containing the accession
                   numbers or other ids of the source sequences to generate the
                   probe
 -e  --extract   flag indicating if accession no. should be extracted from
                   a string of ids separated by the | bar and preceded by a two or
                   three letter code. Takes no argument. Example of the string:
                   ref|NM_178871|gb|AK042509|riken|A630098E12.
                       In this example, the refseq id will be extracted and the
```

```
                   other ids will be discarded
    --source      column number with source name of the sequence accession
                  (E.g. genebank, ensembl, etc) Optional
    --other       other id column
-v  --vgene      column number (starting from 1) containing gene id
                  provided by the vendor
    --refseq      column with RefSeq ids (optional)
    --annotate  Should annotate the probes? (default: "YES")
    --delimiter  character separating columns (default "\t")
    --missing   character for missing observations (default "")
    --recsep      character separating multiple records in a single column field
                  Only the first record is used for annotation but multiple can
                  be are stored in the database (default ";")
-o  --outname   output filename. String with a filename. If given, no
                  changes to the database will be made and am annotated file
                  will be created.
-u  --ugenes    unique-genes output. Returns a list of annotated unique
                  genes. Only used if a text file is the output.
-s  --server     database server [optional]
-d  --database  gene annotation database name [optional]
-u  --user       user name to access database
     --password  password to access database
-h  --help       print this help
```

import_genexref

usage: import_genexref [-options] -i <input filename>

This program will parse a column text file, extract the gene id and some other identification for cross reference and insert this pair in a MySQL database.

```
options
    --input       name of input file with mapping data
    --ignore      number of lines to ignore (default=0)
    --gene_id     column number with gene ids (must be Entrez Ids)
    --xref        column number with cross reference ids
    --delvers     should I delete the version number from the
                  sequence id? (E.g. AV089821.1 -> AV089821)
                  Numeric arguments  0: No, 1: Yes (default=1)
    --type        type of xref ids in the input (string)
    --filter      column number with filter
    --regexp      should I use regular expressions to match filter?
                  (option with no argument)
    --fvalue      value allowed in the filer column
    --delete      column number with gene ids to delete from the db
    --server      database server [optional]
    --user        user name to access database
    --password    user password
    --database    database name
    --delimiter   character separating columns (default '\t')
    --recsep      character delimiting multiple values with a field
    --missing     character for missing values (default: “”)
    --help        print this help
```

import_genemap

usage: import_genemap [-options] -i <input filename>

This program takes as input a column text file of genes and their genomic positions and stores the information in a MySQL database.

options

--input	name of input file with mapping data
--align	title for mouse alignment (short alphanumeric string)
--new	is this a new alignment?
--delimiter	character separating columns (default '\t')
--ignore	number of lines to ignore (default=0)
--gene_id	column number with gene ids (must be Entrez Ids)
--name	column number with gene or sequence name
--chr	column number with chromosome (e.g. 'Chr2')
--strand	column number with strand ('+' or '-')
--txstart	column number with transcription start
--txend	column number with transcription end
--server	database server [optional]
--maptable	table in the database with mapping information
--user	user name to access database
--password	user password
--missing	character for missing values (default: '.')
--help	print this help

accession2gene

usage: accession2gene [-options] -i <input filename>

This program will parse a column text file, extract probe annotations such as accession numbers, RefSeq ids, Ensembl transcripts, gene symbols among other and it will do its best to find the target gene for that probe by accessing a MySQL database. Finally, it will create an annotated output file with Entrez Gene ids and type of sequence used to find it at the last to columns of the file.

options

-i --input	name of input file
--names	first line contains column name (no argument)
-a --accession	column number (starting from 1) containing the accession numbers or other ids of the source sequences to generate the probe Required if no --gene argument is given!
-e --extract	flag indicating if accession no. should be extracted from a string of ids separated by the | bar and preceded by a two or three letter code. Takes no argument. Example of tstring: ref|NM_178871|gb|AK042509|riken|A630098E12. In this example, the refseq id will be extracted and the other ids will be discarded
--other	other id column
-g --genes	column number (starting from 1) containing Entrez Gene IDs Required if no --accession is given!
--refseq	column with RefSeq ids (optional)
--delimiter	character separating columns (default " ")
--missing	character for missing observations (default ".")
--recsep	character separating multiple records in a single column field Only the first record is used for annotation but multiple can be are stored in the database (default ";")
-u --ugenes	unique-genes output. Returns a list of annotated unique genes. Only used if a text file is the output.
-s --server	database server [optional]
-d --database	database name [optional]
-u --user	user name to access database
-o --output	name for output file (default=<intput file>.out)
-h --help	print this help

import_geneinfo

usage: import_geneinfo [-options] -i <input filename>

This program will parse a column text file, and find the gene id and any information related with it and will import it to the ArrayGene database

options

--input	name of input file with mapping data
--new	is this a new alignment?
--ignore	number of lines to ignore (default=0)
--gene_id	column number with gene ids (must be Entrez Ids)
--xref	column number with cross reference ids
--type	type of xref ids in the input (string)
--filter	column number with filter
--fvalue	value allowed in the filer column
--delete	column number with gene ids to delete from the db
--server	database server [optional]
--user	user name to access database
--password	user password
--database	database name
--delimiter	character separating columns (default ' ')
--recsep	character delimiting multiple values with a field
--missing	character for missing values (default: '')
--help	print this help

probe2genemap

usage: probe2genemap [-options] -i <input filename>

This program will parse a column text file, extract probe names for a given microarrays, look for the target gene in the ArrayGene database and retrieve its genomic position in a given genomic assembly. The output is an annotated file with genomic position for every probe (when available).

```
options
 -i  --input       name of input file
     --array       name of microarray
     --probes      column number containing probe identifiers
     --ignore      number of headers lines to ignore (default '0')
     --headers     first line (after <ignore> lines) contain headers
     --symbol      Include gene symbol (Yes: 1, No: 0, Default: '1')
     --gene        Include Entrez Gene id (Yes: 1, No: 0, Default: '1')
     --delimiter   character separating columns (default '	')
     --missing     character for missing observations (default '')
 -s  --server      database server [optional]
 -g  --gdatabase ArrayGene database name [optional]
      --adatabse  Genome Assembly database [optional]
 -u  --user        user name to access database
     --output      name for output file (default=<intput file>.out)
 -h  --help        print this help
```
